# Applications of DNA Hydrogels in Osteoporotic Bone Defects

**DOI:** 10.3390/jfb17080415

**Published:** 2026-08-18

**Authors:** Jiaqi Chen, Huiyu Jia, Xinyue Zhang, Da Liu, Xushuang Jia, Xintong Gu, Hongjuan Wen, Ye Jin

**Affiliations:** 1 School of Pharmacy, Changchun University of Chinese Medicine, Changchun 130117, China; 24204702023@stu.ccucm.edu.cn (J.C.); zhangxy0972@ccucm.edu.cn (X.Z.); liuda@ccucm.edu.cn (D.L.); 23203070205@stu.ccucm.edu.cn (X.J.); 24204893104@stu.ccucm.edu.cn (X.G.); 2 School of Nursing, Changchun University of Chinese Medicine, Changchun 130117, China; jiahuiyu@ccucm.edu.cn; 3 Public Laboratory Centre, Changchun University of Chinese Medicine, Changchun 130117, China; 4 School of Health Management, Changchun University of Chinese Medicine, Changchun 130117, China; 5 Northeast Asia Institute of Traditional Chinese Medicine, Changchun University of Chinese Medicine, Changchun 130117, China

**Keywords:** DNA hydrogel, osteoporotic bone defects, signaling pathways, controlled drug release, microenvironment remodeling

## Abstract

DNA hydrogels are an emerging class of biomaterials with programmability, biodegradability, biocompatibility, and dynamic responsiveness, enabling precise regulation of osteoblast and mesenchymal stem cell (MSC) proliferation and differentiation, activation of key signaling pathways, and promotion of angiogenesis and bone matrix mineralization. In contrast, conventional bone repair materials exhibit limitations including poor mechanical strength, uncontrollable degradation, and inadequate matching with native bone properties, restricting their application in osteoporotic defect repair. Current osteoporotic defect therapies, mainly anti-resorptive and anabolic agents, remain insufficient for many patients. Here, we propose pure and hybrid DNA hydrogels as novel therapeutic platforms to restore the dynamic balance between bone resorption and formation, thereby enhancing osteogenesis and facilitating bone regeneration and remodeling under osteoporotic conditions. Although challenges such as high production cost and long-term safety persist, integration with advanced technologies (e.g., 3D printing and gene editing) may provide theoretical support for further investigation of personalized and intelligent therapeutic strategies at the pre-clinical research stage, offering new insights into osteoporotic bone defects and bone tissue regeneration.

## 1. Introduction

Bone is a critical and dynamic tissue in the human body that responds to mechanical stress and protects internal organs, while playing essential roles in bone metabolism and hematopoiesis. Its primary cellular components include osteoblasts, osteoclasts, bone lining cells, and osteocytes, which function coordinately to maintain the structural integrity of the skeletal framework [1,2]. However, bone tissue damage is highly prevalent in clinical settings. Various conditions, including fractures, osteoporotic bone defects, bone tumors (BTs), and congenital disorders, can lead to skeletal impairment [3,4,5]. It has been reported that millions of patients worldwide receive treatment annually for bone-related disorders, and this number continues to rise each year [6,7]. The healing process of osteoporotic bone defects is highly complex, involving inflammatory responses and cellular proliferation and differentiation that occur in an interconnected and overlapping manner [8,9,10]. During the inflammatory phase, the body initiates an immune response to injury and removes necrotic tissue [11]. During the proliferative and differentiation phase, osteoblasts (OBs) and mesenchymal stem cells (MSCs), among others, play pivotal roles in osteogenesis [12,13]. Osteoporosis is a chronic metabolic bone disease characterized by an imbalance between osteoclast-mediated bone resorption and osteoblast-driven bone formation [14]. Within the pathological microenvironment, osteoporosis is primarily manifested by excessive activation and increased formation of osteoclasts, suppression of osteogenic activity, and concomitant impairment of angiogenesis [15].

Traditional bone repair strategies primarily include autologous bone grafting and allogeneic bone grafting [16,17]. Autologous bone grafting is regarded as the gold standard for the treatment of bone defects due to its excellent osteoinductive, osteoconductive, and osteogenic properties, and it serves as a benchmark for evaluating the performance of other bone grafts and substitutes [18]. Osteoinduction refers to the ability to induce MSCs to differentiate into OBs, thereby promoting new bone formation [19,20]. Osteoconduction provides a structural scaffold that supports the ingrowth of new bone tissue [21]. Osteogenicity denotes the capacity of OBs to secrete various bioactive factors that precisely regulate bone formation and remodeling [22]. However, autologous bone grafting has notable limitations, including limited donor bone availability and restricted harvest volume. Repeated harvesting procedures may increase patient discomfort and surgical trauma, leading to complications such as donor-site pain, infection, hematoma, and nerve injury [23,24]. These complications not only prolong the recovery period but may also result in partial functional impairment at the donor site, thereby negatively affecting patients’ quality of life [25]. Although allogeneic bone grafting alleviates the issue of limited donor supply, it faces the challenge of immune rejection [26,27]. In addition, allogeneic grafts carry risks of disease transmission and bacterial infection, including potential transmission of hepatitis viruses and human immunodeficiency virus (HIV) [28,29]. Given the risks and challenges associated with current bone grafting approaches, there is an urgent need to develop more effective and safer strategies for bone regeneration [30,31].

DNA is a natural, non-toxic, and biodegradable biopolymer. Owing to its excellent biocompatibility, bioresorbability, hydrophilicity, and structural anisotropy, it has emerged as an ideal building block for hydrogel fabrication [32,33]. Previous studies have reported that DNA extracted from pathogens and endogenous fungal mycelia typically consists of fragments larger than 10 kb, exhibiting high stability, high extraction purity, and no residual toxic reagents [34]. Its long-chain structure satisfies the template length requirements for DNA hydrogel formation, while the intrinsic nucleotide composition supports crosslinking modifications. Through physical or chemical crosslinking, DNA can form a three-dimensional hydrogel network that meets both the structural and functional demands of hydrogel-based materials, thereby serving as a feasible construction unit for hydrogel engineering. As an emerging biomaterial, DNA hydrogels have attracted increasing attention in the field of osteoporotic bone defects in recent years and can be broadly classified into pure DNA hydrogels and hybrid DNA hydrogels based on their compositional characteristics [35,36].

Pure DNA hydrogels refer to three-dimensional network structures formed exclusively by DNA strands through complementary base pairing, without the need for additional crosslinkers or synthetic polymers [37,38]. As hydrogels are entirely composed of DNA chains, they can establish stable architectures via hydrogen bonding, physical entanglement, or enzyme-mediated reactions [39]. Owing to their structural stimuli responsiveness and biodegradability, pure DNA hydrogels have emerged as important materials in biomedical applications. These characteristics enable pure DNA hydrogels to closely mimic the structure and function of the natural extracellular matrix (ECM), thereby providing an optimal microenvironment for cell adhesion, proliferation, and differentiation, effectively promoting bone tissue regeneration and repair [40,41]. Moreover, pure DNA hydrogels can serve as carriers for drugs, growth factors, and cells, allowing precise regulation of the bone regeneration process and offering novel strategies for the treatment of osteoporotic bone defects.

Hybrid DNA hydrogels are formed by physically or chemically assembling biomass-derived DNA with other polymers [42,43]. Hybrid DNA hydrogels have demonstrated positive effects on enhancing mechanical strength, improving in vivo stability, and increasing cost-effectiveness [38]. The unique structure of hybrid DNA hydrogels endows them with numerous superior properties, including programmability, excellent stability and flexibility, good biocompatibility and biodegradability, controllable stimuli responsiveness, ease of synthesis, and efficient modifiability [39,43].

**Figure 1:**
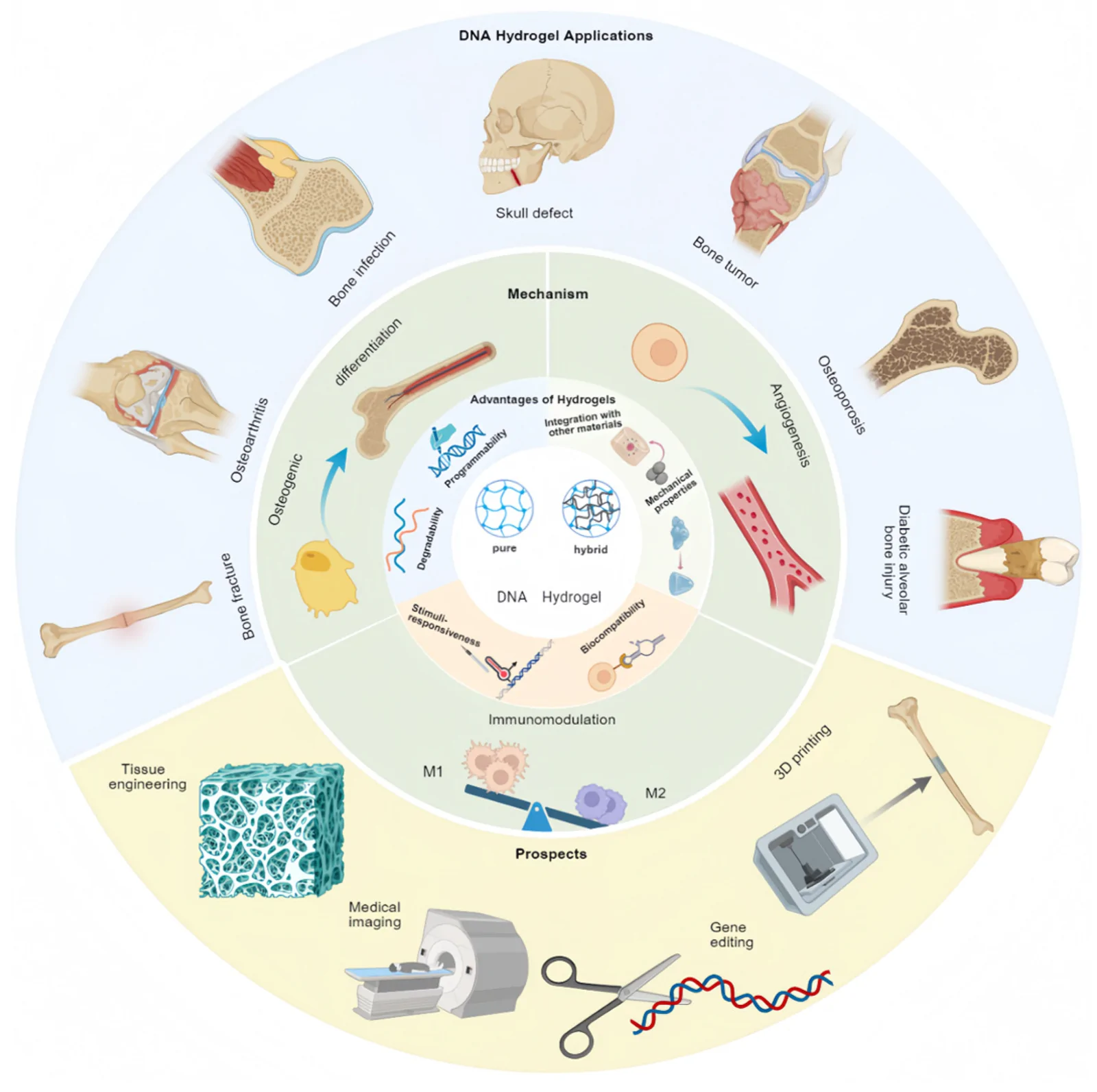
DNA Hydrogels: Properties, mechanisms and applications. This circular overview summarizes diverse bone disease scenarios applicable to DNA hydrogels (including skull defects, bone tumors, osteoporosis, bone infection, fractures, osteoarthritis, diabetic alveolar bone injury) and core functional mechanisms covering osteogenic differentiation, angiogenesis and immunomodulation, as well as forward-looking directions such as 3D printing, gene editing, tissue engineering and medical imaging translation.

Currently, several challenges remain for the application of DNA hydrogels in osteoporotic bone defects, such as the requirement for expensive equipment and specialized techniques, difficulties in ensuring product consistency and stability, and limitations in fully replicating the complex mechanical properties of native bone tissue. However, integrating DNA hydrogels with interdisciplinary technologies, such as gene editing, 3D printing, biosensing, and nanotechnology, can greatly overcome these challenges. Owing to their excellent performance, DNA hydrogels exhibit unique advantages in osteoporotic bone defects and microenvironmental regulation. In-depth investigation of DNA hydrogel applications in osteoporotic bone defects not only facilitates the advancement of bone regenerative medicine and improves therapeutic outcomes for patients but also provides essential theoretical and technical support for related disciplines, including biomaterials science and tissue engineering. Furthermore, DNA hydrogels display prominent research value in the field of regenerative medicine based on current in vitro and small animal results, while relevant human clinical verification is still absent. To visually present the application logic and core value of DNA hydrogels in bone-related diseases, an overview is provided in a graphical abstract format, as shown in Figure 1.

## 2. Properties and Advantages of DNA Hydrogel

The fundamental building block of pure DNA hydrogels is the DNA strand, which can be designed into various modules and patterns based on complementary base-pairing principles (A–T and G–C specific pairing) to achieve self-assembly and form complex, stable three-dimensional network structures [37,44]. Gao and colleagues reported that increasing the length of DNA strands or the proportion of base pairs can enhance the crosslinking density of the hydrogel, thereby improving its mechanical strength [39].

The fundamental building blocks of hybrid DNA hydrogels are hydrophilic polymer chains, which are crosslinked with DNA strands through physical or chemical methods [36]. Amino groups in DNA can react with aldehyde-bearing polymers to form Schiff base linkages, enabling the design of pH-responsive DNA hydrogels [45]. Meanwhile, the negatively charged phosphate groups on the surface of DNA molecules can interact with positively charged compounds, such as chitosan quaternary ammonium salts (Macklin Biochemical Co., Ltd., Shanghai, China), thereby endowing the hydrogel with unique functional properties. Due to the tunable and programmable nature of their structure and properties, pure DNA hydrogels can be engineered with specific functional modifications as needed, resulting in more stable architectures and fully leveraging the advantages of the polymer. These characteristics endow DNA hydrogels with a range of superior properties, offering broad potential for applications in bone tissue regeneration.

Through the integration of DNA with other polymers, hybrid DNA hydrogels can achieve synergistic effects, often described as “1 + 1 > 2.” Owing to the outstanding properties of pure DNA hydrogels and hybrid DNA hydrogels, they provide a platform for biomedical applications such as osteoporotic bone defects.

### 2.1. Properties of DNA Hydrogels

Programmability is a prominent feature that distinguishes hybrid DNA hydrogels from conventional hydrogels [36]. Programmability refers to the rational design of DNA sequences according to specific application requirements, allowing precise adjustment of the structure and functions of DNA hydrogels [46]. In targeted therapy, the incorporation of specific nucleic acid aptamer sequences enables the hydrogel to selectively recognize and bind target cells or biomolecules, thus constructing target-encoded DNA hydrogels for precise therapeutic interventions [47]. In the field of cancer therapy, Zhang and colleagues designed injectable DNA hydrogels loaded with anticancer drugs, which could selectively target tumor cells and significantly enhance therapeutic efficacy [48]. Regarding osteoporotic bone defects, programmable DNA nanostructures and components can precisely regulate the mineralization of calcium phosphate, effectively guide bone tissue formation, and accelerate bone remodeling [35,49]. The sequence programmability of DNA hydrogels also enables specific capture and delivery of complex bioactive payloads through precise molecular design, facilitating the construction of smarter therapeutic systems. GS/sEV@DNAgel developed by Xing et al. leverages the programmable properties of DNA hydrogels. DNA aptamers that specifically recognize CD63 (Cluster of Differentiation 63), a surface marker on small extracellular vesicles (sEVs), are incorporated into its network structure, achieving high-affinity and specific binding between aptamers and antigens [50].

DNA hydrogels exhibit stimuli-responsiveness to factors such as temperature, pH, ionic strength, and biomolecules [51]. These responsive behaviors enable the hydrogels to undergo conformational changes in response to environmental variations, dynamically modulating their macroscopic properties and thereby facilitating controlled drug release and regulation of cellular behavior [44,52]. Upon temperature changes, certain thermoresponsive DNA hydrogels undergo reversible phase transitions at their critical solution temperature, allowing precise control over drug release rates [53,54]. When the microenvironmental pH fluctuates at the lesion sites of osteoporotic bone defects, pH-responsive DNA hydrogels respond accordingly, promoting drug release and enhancing therapeutic efficacy [55].

**Figure 2:**
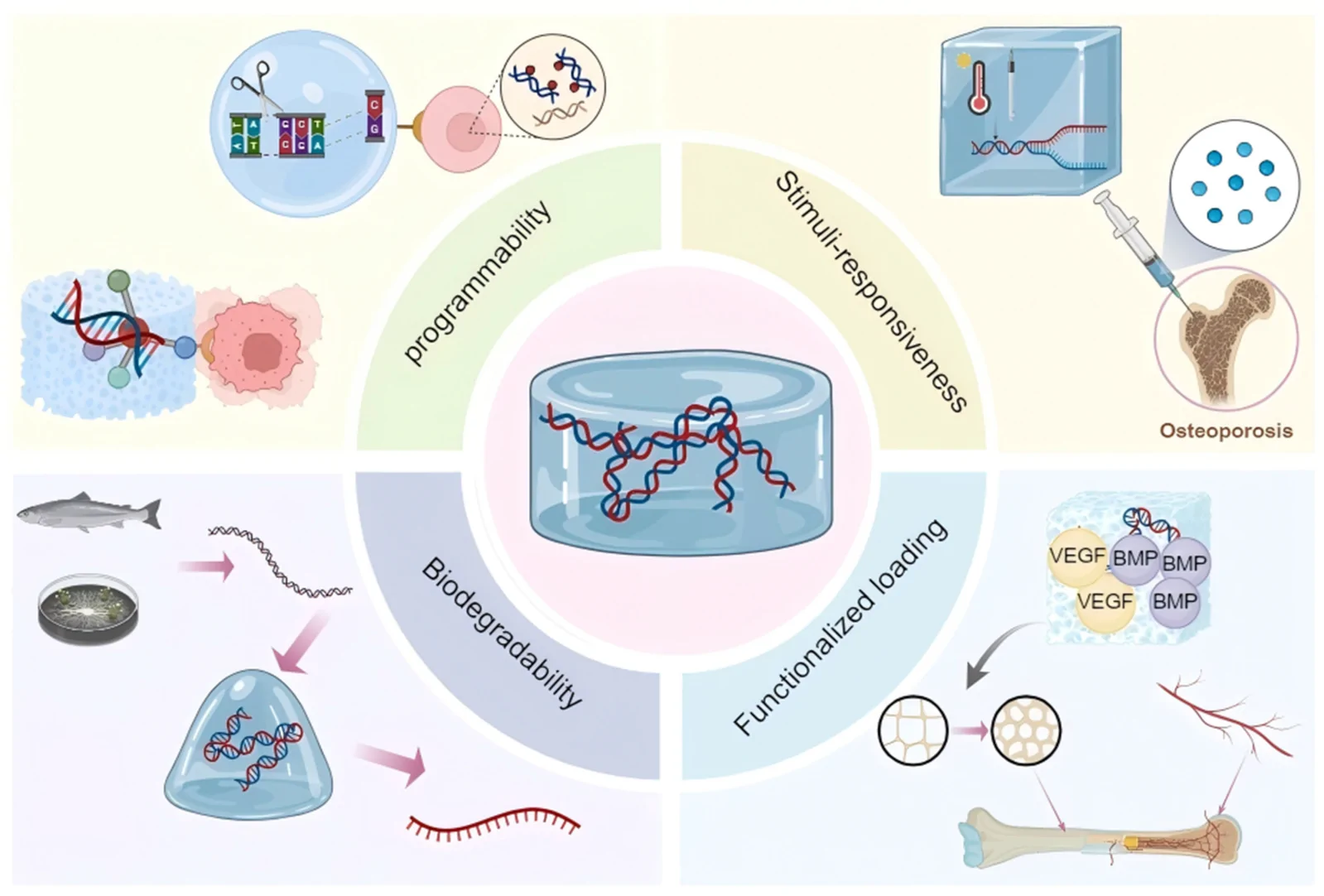
Key properties and applications of DNA hydrogels.

DNA is a natural molecule within living organisms; therefore, DNA hydrogels exhibit excellent biocompatibility and generally do not elicit severe immune responses [56]. Nucleotides, as the building blocks for synthetic DNA, participate in cellular metabolic activities and promote the proliferation and differentiation of OBs [57,58]. In vitro studies have shown that OBs cultured on DNA hydrogels demonstrate high cell viability and proliferation capacity [35,59]. In vivo experiments similarly indicate that implanted DNA hydrogels exhibit favorable biocompatibility, providing strong support for tissue regeneration [60]. Collectively, both in vitro and in vivo studies demonstrate that DNA hydrogels can support normal cellular metabolism and function, show negligible cytotoxicity, and exert beneficial effects during osteogenesis. DNA hydrogels undergo safe biodegradation, yielding small molecules such as nucleotides that can be metabolized and absorbed by the body, thereby avoiding potential risks associated with long-term implantation [61,62]. Moreover, the self-healing properties and injectability of DNA hydrogels make them suitable for minimally invasive therapies, offering a multifunctional and customizable approach to bone remodeling. In terms of physical properties, DNA hydrogels enable cascade regulation of the immunity–angiogenesis–osteogenesis axis via tunable pore sizes: they simultaneously induce the polarization of macrophages toward the anti-inflammatory M2 phenotype and facilitate the proliferation of vascular endothelial cells. By modulating crosslinking density, DNA hydrogels can be tailored to possess an elastic modulus matching that of osteoporotic bone, thereby mitigating the stress shielding effect. DNA hydrogels self-assemble into three-dimensional porous architectures that closely recapitulate the structure of the native extracellular matrix (ECM). Benefiting from this structural similarity, they are capable of recapitulating the physical and chemical cues delivered by the native ECM to cells. Their hierarchical hydrophilic pores and dynamic network structure, which mimic those of ECM, establish efficient transport pathways between cells and the matrix for nutrients, ions and growth factors, ultimately boosting osteogenic differentiation [63]. Future work should focus on optimizing degradation kinetics and long-term safety to facilitate clinical translation. Based on the key properties of DNA hydrogels, Figure 2 provides a visual overview of the correspondence between their characteristics and potential application scenarios.

The diagram systematically exhibits four intrinsic advantages of DNA hydrogels, including programmability, stimuli-responsiveness, biodegradability and functionalized modification. These characteristics support gene-editing manipulation, lesion microenvironment-responsive targeted delivery, eco-friendly raw material extraction and in vivo degradability, as well as structural optimization to facilitate angiogenesis and osteogenic regeneration for osteoporotic bone defect therapy.

### 2.2. Advantages of DNA Hydrogels Compared to Traditional Materials

Conventional biomaterials used for bone repair primarily include natural and synthetic biodegradable polymers, ceramics (including bioactive glass), metallic materials, and polymeric hydrogels [64]. Among these, titanium, a commonly used metal in orthopedic applications, exhibits excellent mechanical properties; however, its implantation may lead to the release of titanium ions, potentially exacerbating inflammation in surrounding tissues and interfering with normal cellular functions [65,66]. Ceramic materials, such as hydroxyapatite, are among the most widely applied biomaterials for bone repair. While they possess certain osteoconductive and bioresorbable properties, they suffer from poor toughness, limited resorbability, and restricted bioactivity [67,68]. Existing polymeric hydrogels include natural polymers (natural polysaccharides and proteins), synthetic polymers (artificially chemically synthesized), and composite polymers [69]. Natural polymeric hydrogels with wide raw material sources, superior biocompatibility and low immunogenicity lack sufficient mechanical strength for weight-bearing bone defects. Synthetic hydrogels possess customizable mechanical and degradable properties but bear potential cytotoxic risks over long-term implantation. Composite hydrogels combine the bioactivity of natural polymers and robust mechanical performance of synthetic polymers, while the asynchrony between degradation and drug release and complicated multi-component manufacturing remain major drawbacks. Compared with traditional bone regenerative materials, DNA hydrogels combine the advantages of both DNA and hydrogel systems, demonstrating superior potential in bone tissue regeneration. In terms of biocompatibility, DNA hydrogels exhibit favorable bioactivity, further enhancing cell–material interactions and promoting bone tissue regeneration [70]. Studies have shown that DNA hydrogels can provide a favorable microenvironment for cells by reducing immune responses and simultaneously promoting OB proliferation and differentiation [71]. Traditional bone repair materials still face challenges in precise drug delivery and controlled release. Hybrid DNA hydrogels, by integrating their multifunctional structures with intrinsic biological activity, can overcome these limitations. Through the design of specific DNA sequences, they enable efficient drug loading and controllable release, thereby addressing shortcomings of conventional materials [49,72].

In terms of stimuli-responsiveness, DNA hydrogels can sense changes in the microenvironment at osteoporotic bone defect sites, such as alterations in pH or increases in specific biomolecule concentrations, enabling precise drug release and thereby significantly enhancing therapeutic efficacy [73]. Regarding programmability, hybrid DNA hydrogels utilize their programmable three-dimensional network structures to achieve precise encapsulation of growth factors, which are subsequently released in vivo to sustainably promote bone tissue regeneration [74]. Compared with traditional materials, DNA hydrogels loaded with growth factors can maintain effective concentrations for a longer duration while precisely regulating OB proliferation and differentiation, thereby accelerating the repair of bone defects [75,76]. Traditional materials generally exhibit fixed properties and functions after formation, whereas DNA hydrogels can achieve structural and functional diversity through simple modifications of DNA sequences to meet various clinical needs [77]. Owing to their flexible programmability, DNA hydrogels can be tailored to different anatomical sites and severities of osteoporotic bone defects, with tunable mechanical properties and degradation rates, thereby improving the precision and efficacy of bone defect repair observed in in vitro and small animal experimental models [78,79]. For load-bearing sites, such as the femur, where higher mechanical strength is required, DNA hydrogels with elevated mechanical properties and slower degradation rates should be designed to provide stable support during bone regeneration [77,80]. Conversely, for non-load-bearing small defects, such as those in the cranial bone, lower mechanical strength is sufficient, while faster repair and integration with surrounding tissues are prioritized. In these cases, DNA hydrogels with lower mechanical strength and faster degradation rates are more appropriate. Furthermore, the drug release profiles of DNA hydrogels can be precisely engineered based on factors such as local inflammation levels and growth factor requirements at the lesion site [78].

DNA hydrogels exhibit significant overall advantages over traditional bone repair materials in terms of mechanical adaptability and degradation safety. Regarding mechanical performance, conventional metallic materials often display mechanical properties that do not match those of native bone tissue, which can induce stress shielding effects and negatively affect normal bone regeneration and remodeling [81]. For example, the elastic modulus of titanium alloys is much higher than that of human bone, reducing the mechanical load on bone, increasing bone resorption, and impairing bone healing [82]. Ceramic materials, on the other hand, are highly brittle and prone to fracture under substantial external forces [83]. Natural polymer-based hydrogels generally suffer from inferior mechanical properties. Their overall elastic modulus, tensile strength and compressive strength are far lower than those of human cortical bone, resulting in poor load-bearing capacity, soft texture, and susceptibility to breakage and collapse [84]. In contrast, hybrid DNA hydrogels demonstrate tunable mechanical properties; by adjusting DNA sequences and crosslinking density, they can potentially achieve favorable mechanical matching with native bone tissue [85]. Studies have incorporated specific nanomaterials or biomolecules into hydrogels to enhance mechanical performance while maintaining excellent biocompatibility and bioactivity [86]. Regarding biodegradability, some traditional repair materials face difficulties in degradation and may generate unsafe degradation products. For instance, certain polymeric materials degrade slowly in vivo, potentially leading to long-term residue and eliciting inflammatory responses [87]. In the degradation of DNA hydrogels, the products are small molecules such as nucleotides, which can be metabolized and absorbed by the body, posing no adverse effects on surrounding tissues and exhibiting high safety. Moreover, during bone remodeling, these degradation products can participate in normal metabolic processes, providing essential material support for new bone formation [88]. This feature renders DNA hydrogels particularly advantageous in osteoporotic bone defect applications, reducing potential risks and concerns for patients [58,89].

The following summarizes the performance comparison between traditional bone repair materials and DNA hydrogels, highlighting that programmability represents a unique advantage of DNA hydrogels, as shown in Table 1.

## 3. DNA Hydrogels for Osteoporotic Bone Defects

Osteoporotic bone defect formation is a complex and continuous process precisely coordinated by various endogenous factors [111]. Common components include cellular constituents, growth factors, and signaling pathways, which collectively function to repair damaged bone tissue [120]. Bone remodeling is driven by multiple cell populations working synergistically within a complex network of signaling regulation [121]. At the cellular level, the main participants include OBs, osteoclasts (OCs), and macrophages.

### 3.1. Regulation Mechanisms of Osteoporotic Bone Defects Based on Bone Remodeling Under Multiple Cells and Growth Factors

Bone remodeling is achieved through a dynamic balance between osteoblast-mediated bone formation and osteoclast-driven bone resorption, ultimately resulting in structurally mature and functionally stable bone tissue [122]. The primary cause of osteoporotic bone defects is the disruption of this bone homeostasis.

Osteogenesis begins with the differentiation of MSCs into the osteoblastic lineage, proceeding through a pre-osteoblast (pre-OB) intermediate stage; mature osteoblasts synthesize and deposit bone matrix proteins, thereby promoting bone formation [123]. MSCs can be isolated from various tissues, among which bone marrow-derived mesenchymal stem cells (BMSCs, ScienCell Research Laboratories, Carlsbad, CA, United States, Cat. No. R7500) are one of the most widely applied due to their distinct advantages [124]. BMSCs were first identified as multipotent cells in the bone marrow, possessing the potential for multilineage differentiation, self-renewal, and immunomodulation and capable of differentiating into osteoblasts, chondrocytes, adipocytes, and other cell types, playing crucial roles in tissue engineering and regenerative medicine [125]. Studies have shown that DNA hydrogels can specifically interact with BMSCs to construct a protective microenvironment, effectively buffering shear stress and thereby delaying the pathological progression of osteoarthritis (OA). Miao et al. developed a macroporous double-network DNA hydrogel, which provides nucleic acid aptamers and nano-inducers, enhancing BMSC recruitment, promoting differentiation toward the osteogenic lineage, and accelerating bone remodeling. DNA hydrogels can promote BMSC adhesion and osteogenic differentiation, likely due to enhanced electrostatic interactions between the negatively charged glycoproteins and proteoglycans on the cell surface, which activate intracellular osteogenesis-related signaling pathways [126,127].

OBs are the functional cells responsible for bone regeneration, with a core role in synthesizing and secreting the organic bone matrix and guiding its mineralization, a process that forms the biological foundation for skeletal development and lifelong bone remodeling [128]. The osteogenic-promoting potential of DNA hydrogels may originate from their phosphate-rich backbone structure and adenine content [35]. Adenine can enhance OB differentiation under both normal and inflammatory conditions, upregulating collagen gene transcription and increasing alkaline phosphatase (ALP) activity [129]. ALP activity is an early marker of OB differentiation; its enhancement directly accelerates OB differentiation and activation, further progressing them toward maturation [130]. Studies have shown that as a three-dimensional growth scaffold, DNA hydrogels can maintain OB viability in vitro while promoting their osteogenic differentiation in vivo [35].

During bone remodeling, OCs are responsible for bone resorption, adhering to bone surfaces and degrading the bone matrix to effectively remove old or damaged bone, which is essential for maintaining bone health and homeostasis [131,132]. In a rat calvarial model, DNA hydrogels inhibited OC activity at 10 days post-surgery, protecting new bone mineralization; by 28 days post-surgery, moderate OC activity was maintained to match the needs of bone tissue remodeling, collectively supporting the dynamic balance of bone defect healing. These findings suggest that the regulatory balance between osteoblast and osteoclast activity is a fundamental determinant of bone repair capacity [133].

Immune cells play an auxiliary role in bone regeneration. Among the cells of the immune system, macrophages are one of the most extensively studied cell types, serving not only as a core component of the tissue regeneration microenvironment but also exerting regulatory effects on BMSCs [134]. Chu et al. [135] indicated that the close interaction between BMSCs and macrophages plays a key role in maintaining bone tissue homeostasis. Related studies have also confirmed that macrophages possess the potential and capability to regulate the osteogenic differentiation of BMSCs [136].

Bone formation is a multifactorial and synergistically regulated process, requiring the coordinated participation of multiple bone growth factors [137]. For instance, the synergistic effects of bone morphogenetic proteins (BMPs), vascular endothelial growth factor (VEGF), and platelet-derived growth factor (PDGF) have been validated in various animal models of osteoporotic bone defects [138]. Transforming growth factor-β (TGF-β) is a multifunctional growth factor with dual osteogenic and osteoinductive activities, which plays critical roles in multiple biological processes including cell growth, differentiation, apoptosis and immune responses [139]. As a core member of the TGF-β superfamily, BMPs are multifunctional growth factors with both osteogenic-promoting and bone-inducing functions [140,141]. Their primary role is to induce the proliferation of MSCs and other osteoprogenitor cells and to direct their differentiation into OBs, thereby initiating osteogenesis across multiple cell types [140,142]. In a cell study using DNA hydrogels loaded with BMP-2 (Bone Morphogenetic Protein-2), BMP was shown to effectively promote the proliferation and differentiation of rat MSCs, while histochemical staining indicated increased expression of ALP and osteocalcin (OCN) [143]. BMP-loaded DNA hydrogels can enhance ALP activity in OBs and significantly promote OB differentiation and bone mineralization. Bai et al. demonstrated that a novel nanocomposite system composed of tetrahedral framework nucleic acids (tFNAs) complexed with quercetin (tFNAs/Que) promoted the early expression of osteogenic markers such as Runx2 (Runt-related Transcription Factor 2) and Osterix, thereby enhancing BMSC osteogenic differentiation [144].

**Figure 3:**
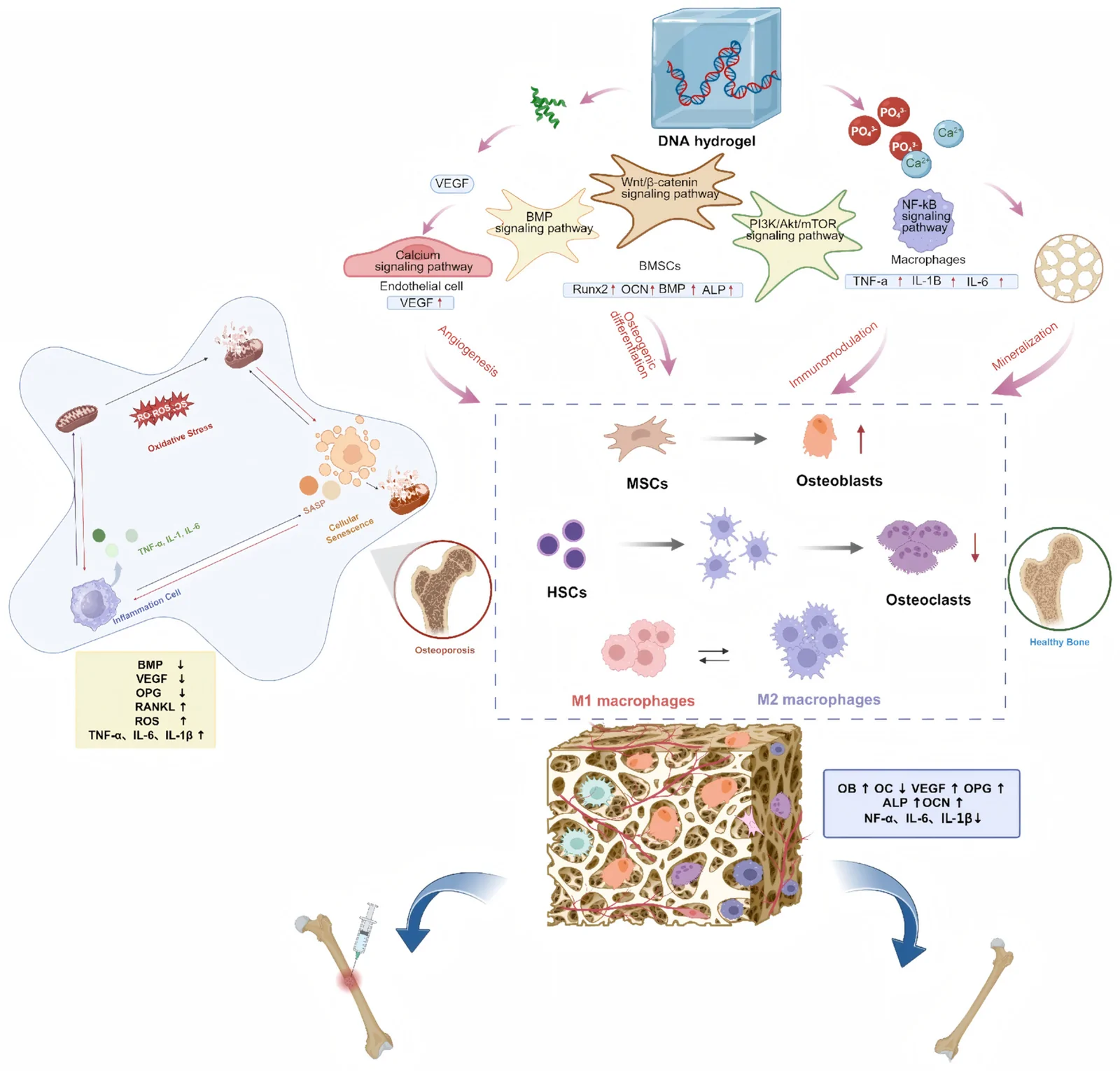
Schematic diagram of the mechanism by which DNA hydrogels regulate bone regeneration.

Given the synergistic coupling between osteogenesis and angiogenesis, angiogenesis-related cytokines (PDGF, VEGF) play an indispensable regulatory role in the bone regeneration process [145]. VEGF acts as a critical coordinator in the growth plate, regulating chondrocyte apoptosis, hypertrophic chondrocyte function, extracellular matrix remodeling, angiogenesis, and bone formation. In DNA hydrogels loaded with VEGF, precise release and synergistic effects play key roles in the promotion of angiogenesis [76]. Based on the above, the core regulatory mechanisms of DNA hydrogel-mediated bone regeneration and the interactions among various factors are illustrated in Figure 3.

After implantation into osteoporotic bone defects, DNA hydrogels exert synergistic regulatory effects on angiogenesis, osteogenic differentiation, immune microenvironment remodeling and extracellular matrix mineralization via multiple signaling pathways. It relieves excessive inflammation and oxidative stress, balances the activity of osteoblasts and osteoclasts, rectifies the disturbed bone metabolism under osteoporotic pathological conditions, and ultimately repairs bone lesions and reconstructs normal bone tissue structure.

### 3.2. Regulation Mechanisms of Key Signaling Pathways in Bone Regeneration Under Osteoporotic Bone Defects

During bone regeneration and remodeling, multiple signaling pathways act in a coordinated manner to finely regulate the proliferation and differentiation of OBs and to precisely control the synthesis and mineralization of the bone matrix they mediate [146]. This review summarizes that DNA hydrogels, via controlled delivery of drugs and osteogenic growth factors, achieve targeted regulation of canonical osteogenic signaling pathways including intracellular Wnt/β-catenin, BMP, and PI3K/Akt/mTOR, thereby modulating bone remodeling processes.

The Wnt/β-catenin and BMP signaling pathways are two critical pathways regulating bone formation. Wnt signaling includes the canonical β-catenin-dependent pathway and non-canonical β-catenin-independent pathways [147]. Wnt proteins are secreted and, upon binding to their corresponding receptors, cause β-catenin to accumulate in the cytoplasm and translocate to the nucleus, where it interacts with transcription factors to form a complex that activates the transcription and expression of osteogenic genes—this constitutes the canonical Wnt pathway [148,149]. Non-canonical *Wnt5a* can directly participate in osteoblast differentiation and can also indirectly enhance Wnt/β-catenin signaling by promoting *Lrp5/6* expression through autocrine secretion by OBs [150]. The Wnt/β-catenin pathway is essential for skeletal development, tissue formation, and the maintenance of growth plate and articular cartilage function [151]. Activation of this pathway induces the expression of transcription factors, promoting osteoblast differentiation [150]. Pre-clinical studies in transgenic mice and human studies have shown that abnormal Wnt/β-catenin signaling is a core factor in osteoarthritic disease, acting directly on bone tissue [152]. Ben et al. demonstrated that inhibition of Wnt/β-catenin signaling reduces bone mass, whereas activation promotes bone formation [153]. Moreover, the Wnt/β-catenin pathway plays a key role in regulating angiogenesis [154]. Shen J. et al. [155] reported that the angiogenic factor EGFL6 stimulates vascular formation and osteogenic differentiation in vitro through activation of Wnt/β-catenin signaling, enhancing BMSC osteogenic potential and promoting angiogenesis to accelerate bone regeneration. Zhao et al. [156] found that macrophages, via MSR1-mediated activation of the PI3K/AKT/GSK3β/β-catenin pathway, promote BMSC osteogenic differentiation under co-culture conditions. In a dual-network DNA hydrogel system, nucleic acid aptamers are utilized to achieve targeted recruitment of endogenous bone marrow mesenchymal stem cells (BMSCs). This biomaterial can sustainably release Wnt signaling molecules at the injury site, activate the intracellular osteogenic Wnt/β-catenin signaling axis in BMSCs, drive the directional osteogenic differentiation of stem cells, and ultimately facilitate robust new bone regeneration within the defect area [157]. There are two key inhibitors for the Wnt/β-catenin signaling pathway: sclerostin and DKK-1 [158]. Among them, sclerostin is a glycoprotein associated with bone metabolism regulation that is highly specifically secreted by osteocytes. It binds to LRP5/6 and LRP4, thereby inhibiting the intracellular Wnt/β-catenin signaling pathway in osteocytes [159,160]. DKK-1 belongs to the cysteine-rich protein family. It antagonizes the canonical Wnt pathway by interacting with LRP5/6 and Frizzled and exerts crucial regulatory effects on new-bone formation in bone-related diseases [161].

**Figure 4:**
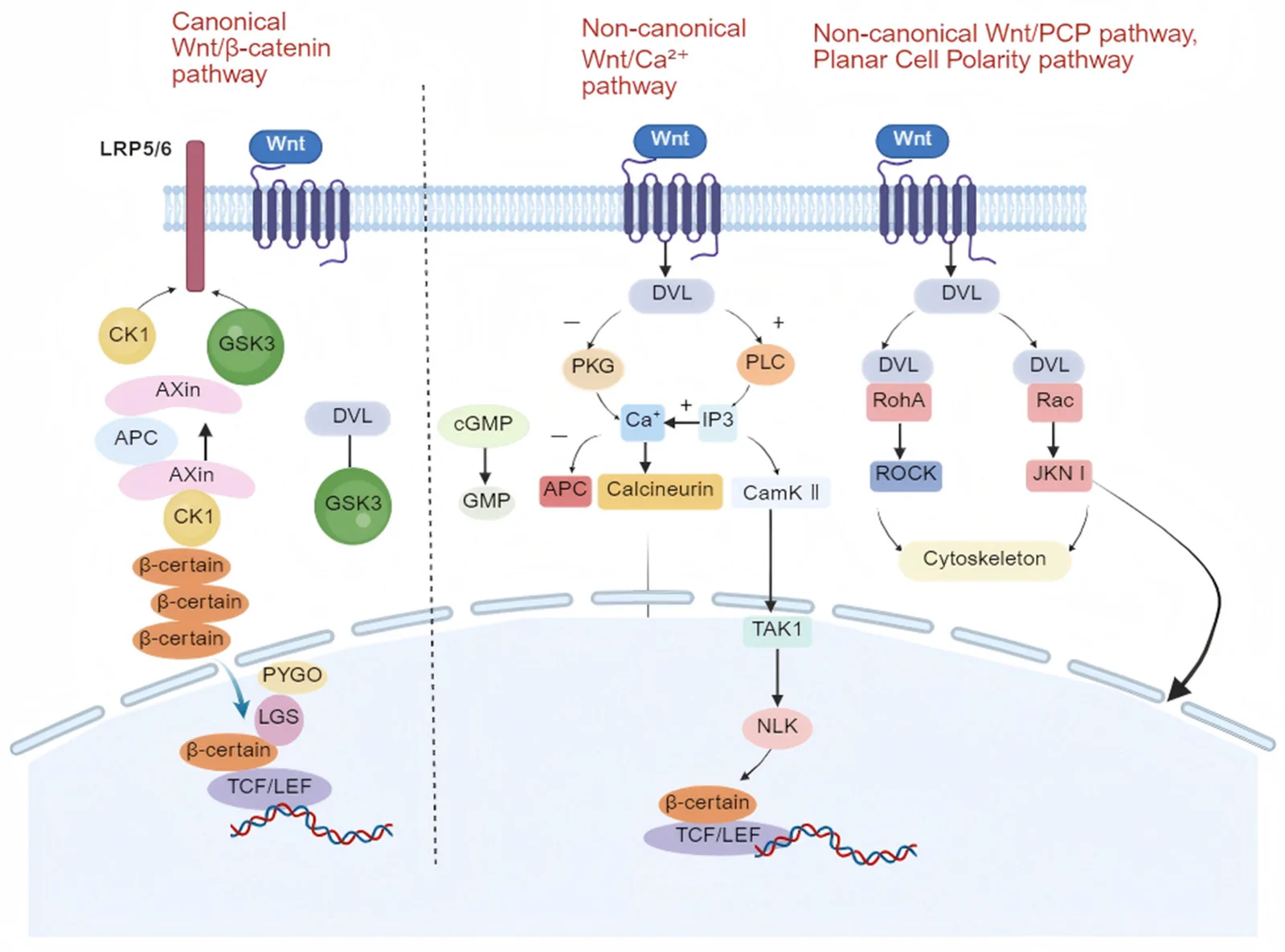
Regulatory mechanism of the Wnt signaling pathway.

As a central regulatory pathway in bone regeneration, the Wnt signaling pathway exhibits distinct but interconnected mechanisms in its canonical and non-canonical routes. Figure 4 illustrates the specific signal transduction processes and molecular mechanisms.

This figure illustrates three major branches of Wnt signaling cascades, including the canonical Wnt/β-catenin pathway, non-canonical Wnt/Ca^2+^ pathway and non-canonical Wnt/planar cell polarity (PCP) pathway. It displays the whole signal transduction process from membrane receptor activation and intracellular cascade transmission to nuclear translocation of downstream transcription factors for target gene transcription.

Currently, in vertebrate skeletal biology, the BMP superfamily signaling pathway has been the most extensively studied [162]. Depending on whether it is Smad-dependent, BMP signaling can be classified into canonical and non-canonical pathways [140]. The BMP signaling pathway begins when BMP molecules specifically bind to type I and type II serine/threonine kinase receptors. This leads to phosphorylation of the glycine–serine (GS) domain in the type I receptor, activating receptor kinase activity. The activated receptor can then transmit signals to the nucleus through two mechanisms: Smad phosphorylation or MAPK (mitogen-activated protein kinase) activation, thereby regulating the expression of osteogenesis-related genes and promoting osteoblast differentiation [163,164]. The non-canonical pathways include MAPK-Smad2/3 and ERK-MAPK cascades induced via BMP-mediated MAPK signaling [165]. Studies have shown that BMP-loaded DNA hydrogels implanted at osteoporotic sites can sustainably release BMP, activate BMP signaling, upregulate osteoblast markers such as ALP and OCN, enhance bone matrix synthesis and mineralization, and drive MSC differentiation toward osteoblasts, thereby accelerating bone tissue remodeling [166,167]. Acting as a porous scaffold, DNA hydrogel delivers BMP to protect the growth factor and realize sustained release. It further activates the BMP/Smad signaling pathway, upregulates osteogenic genes such as Runx2, ALP and OCN, stimulates mesenchymal stem cell *proliferation* and differentiation, and accelerates bone defect repair [157]. During osteogenesis, although the WNT and BMP signaling pathways possess independent signaling systems and can individually regulate osteogenesis via their respective ligands and receptors, as well as intracellular and nuclear signaling factors, they do not function in complete isolation and exert mutual regulatory effects [168]. Studies have revealed that the osteo-inducer Ganoderma-A (GD-A) can markedly upregulate the expression of multiple osteoblastic differentiation markers. It mainly induces the differentiation of human amniotic mesenchymal stem cells into osteoblasts by mediating the crosstalk between the Wnt/β-catenin and BMP/SMAD signaling pathways [169].

The Notch signaling pathway regulates somite segmentation, patterning, and the differentiation of osteoprogenitor chondrocytes, collectively promoting normal axial skeletal development [170]. This pathway serves as a key cell fate determinant during prenatal skeletal development and continues to play a role in maintaining adult tissue homeostasis [171]. Notch ligands interact with receptors via their extracellular domains, triggering a proteolytic cascade that cleaves and releases the Notch intracellular domain (NIC) from the cell membrane [172]. Once released, the NIC translocates to the nucleus with the aid of co-activators (such as transcription factors) and activates downstream target gene expression through both canonical and non-canonical mechanisms [173,174]. Studies have reported that Jagged1, a critical ligand in the Notch pathway, promotes osteogenic differentiation of human periodontal ligament cells by activating Notch signaling [175]. Additionally, activation of the Notch pathway can inhibit β-catenin expression, indicating crosstalk between Notch and Wnt/β-catenin signaling [176]. Specifically, they co-regulate progenitor-cell proliferation and osteogenic differentiation. Notch1 promoter methylation attenuates Notch pathway activity, de-repressing Wnt signaling to boost osteogenic differentiation, while Wnt signaling promotes early osteoblast formation yet suppresses terminal cellular differentiation [177].

The PI3K/Akt/mTOR signaling pathway is a key intracellular signal transduction pathway that plays an important role in bone metabolism and remodeling, widely participating in the regulation of OBs, OCs, and bone marrow mesenchymal stem cells (BMSCs) proliferation and differentiation [178]. Activation of the PI3K/Akt/mTOR pathway begins when membrane-bound class I PI3K is specifically recruited and activated by upstream signals mediated through G-protein-coupled receptors (GPCRs) or receptor tyrosine kinases (RTKs) [179,180]. Activated class I PI3K catalyzes the phosphorylation of phosphatidylinositol 4,5-bisphosphate (PIP2), generating the second messenger phosphatidylinositol 3,4,5-trisphosphate (PIP3) [181]. PIP3 then acts as a second messenger to recruit signaling molecules such as phosphoinositide-dependent kinase 1 (PDK1) to the cell membrane, leading to phosphorylation and activation of Akt [182]. Activated Akt directly stimulates mTORC1, which is a major downstream effector regulating cell growth, metabolic activity, and protein synthesis [183]. Additionally, sustained Akt activation can inhibit apoptosis and promote cell growth, proliferation, and DNA repair [184]. Studies have shown that multifunctional DNA hydrogels encapsulating exosomes can significantly enhance alveolar bone defect repair, possibly through proteins enriched in ASC-EVs activating the PI3K-Akt pathway, while miRNA-150-5p carried by these exosomes further enhances osteogenesis via regulation of this pathway [185]. Studies have demonstrated that an engineered exosome co-modified with CD47 protein and 5HT1D antibody can be fabricated using a nanosecond-pulsed microfluidic system [186]. Such engineering modification endows exosomes with the capability to resist phagocytosis by macrophages and evade immune clearance. These findings suggest that combining DNA hydrogels with the above-mentioned engineered exosomes holds great promise as a novel strategy to address the bottleneck of hampered drug delivery within the pathological microenvironment of osteoporosis.

In dual-signal axis regulation of bone remodeling, functionalized hydrogels with black phosphorus nanosheets have been applied [187]. On one hand, VEGF activates the VEGFR2/PI3K/AKT pathway, promoting endothelial cell migration and lumen maturation via Notch-Dll4, establishing functional vascular networks. On the other hand, phosphate ions released from black phosphorus nanosheet degradation activate the FGF23/Klotho-FGFR1c pathway, upregulating the BMP-2/Smad1/5/8–Runx2 cascade in MSCs, while the ROS/p38 MAPK pathway enhances Osterix transcriptional activity, driving temporal expression of osteogenic markers and directing bone matrix mineralization.

This multi-target, multi-pathway synergy reshapes the microenvironmental interplay between angiogenesis and osteogenesis, overcoming the limitations of traditional materials that rely on single-action pathways. Each signaling pathway plays a core regulatory role in osteogenesis, and activation triggers a cascade of downstream effects. Direct targeting of these pathways allows for precise intervention and optimization of the bone formation process [188].

DNA hydrogels can regulate these key pathways in multiple dimensions. Their molecular mechanisms are systematically summarized in Table 2.

### 3.3. DNA Hydrogels Activate Corresponding Signaling Pathways via Multiple Mechanisms

Both pure DNA hydrogels and hybrid DNA hydrogels can, through molecular loading or surface modification, enhance tissue interactions, achieve slow in vivo release, and simultaneously activate relevant signaling pathways, conferring multifunctional therapeutic properties [190,191]. For example, in osteoporotic bone defects, pro-inflammatory factors such as TNF-α, IL-1β, and ROS significantly increase, while osteogenesis-related factors such as Runx2 and BMP-2 decrease. DNA hydrogels can be loaded with growth factors like BMP-2 and VEGF, activating signaling pathways to restore factor levels and promote bone remodeling.

Another strategy exploits the microenvironment-responsive properties of DNA hydrogels: when the hydrogel senses specific microenvironmental changes at the osteoporotic bone defect site, its structure transforms, releasing signaling pathway activators on demand to achieve targeted pathway activation [60]. Currently, various intelligent responsive hydrogels have been developed, including those responding to endogenous biological signals (enzymes, pH, temperature, ROS) and exogenous physical stimuli (magnetic, electrical, light), allowing them to function according to physiological characteristics or external environmental cues [192].

Hybrid DNA hydrogels use a double-network system, integrating the advantages of multiple components, combining balanced mechanical strength with good bioactivity. Zhou et al. [193] designed a double-network DNA-SF hydrogel that remodels the mechanical microenvironment for cartilage repair by regulating matrix stiffness: the DNA network induces SF (Simatech Biomaterials Co., Ltd., Suzhou, China) to form β-sheets, creating a mechanical gradient; this synergistically activates Wnt/β-catenin and TGF-β/Smad pathways, upregulates cartilage marker genes, directs BMSCs toward chondrogenic differentiation, and ultimately achieves both structural and functional cartilage repair.

Furthermore, DNA hydrogels may indirectly activate signaling pathways through interactions with cell surface receptors. Specific ligands on the hydrogel surface can bind to cell surface receptors, triggering intracellular signaling cascades and ultimately activating Wnt/β-catenin or BMP pathways to promote bone regeneration [194]. Studies have shown that surface modification of DNA hydrogels with bioactive molecules (e.g., collagen, integrins) enhances interactions with surrounding tissues [195,196]. Hao et al. designed a biomaterial using integrin-targeting ligands LLP2A and LXW7 (targeting bone formation-related integrin α4β1 and vascularization-related integrin αvβ3, respectively) to regulate endogenous cell adhesion and promote vascularized bone regeneration. In vitro experiments demonstrated that the material improved adhesion of MSCs, OBs, and endothelial cells via the two integrins; in adult rat cranial and fetal sheep spinal osteoporotic bone defect models, it enhanced bone and vessel formation, highlighting the role of integrins in cell adhesion and bone regeneration [189]. Cellular and animal experiments show that the dual-integrin-targeting strategy of this biomaterial synergistically modulates cell adhesion and achieves coupled angiogenesis–osteogenesis. Nevertheless, the interaction between the two ligands is unclear. The fetal-sheep-bone model cannot fully recapitulate clinical pathological osteoporotic bone defects, and data from mature large-animal models are still needed. Another study [197] developed RGD-modified silk fibroin–DNA double-network hydrogel microspheres (RSD-MSs), which synergistically regulate the integrin α5β1–FAK–PI3K/Akt mechanotransduction pathway through biomimetic ECM topology and dynamic DNA–β-sheet double-network structures. The RGD peptide specifically enhances cell adhesion and activates the SOX9 transcription factor-mediated chondrogenic program. This system simulates the natural cartilage microenvironment, promotes BMSCs to differentiate into functional chondrocytes, upregulates GAG synthase expression, and achieves regeneration of hyaline cartilage-specific ECM. This hydrogel, through physical–chemical–biological multidimensional regulation, remodels the cartilage homeostatic microenvironment and provides an innovative material platform for organ-on-chip-level cartilage organoid construction.

## 4. Clinical Applications of DNA Hydrogels

Beyond various osteoporotic bone defects and bone pathologies, DNA hydrogels show promising potential in multiple pre-clinical scenarios related to bone regeneration and remodeling, with repair strategies validated in experimental models for different types of bone defects and pathogenic causes.

Osteoporotic bone defects are fundamentally caused by systemic bone metabolism disorders, leading to localized pathological damage. These processes are regulated by multiple factors, including hormone levels, cytokines, and nutrient metabolism, and are closely related to deterioration of bone microarchitecture and cellular dysfunction. Traditional bone grafting faces limitations due to donor scarcity and immune rejection, making functional regeneration difficult [198]. Han et al. [60] developed a thermosensitive DNA hydrogel capable of self-assembly at 37 °C via nucleic acid nanotechnology. Its porous structure was loaded with Apt02-functionalized tetrahedral framework nucleic acids (tFNA) to form a multimodal repair system. Apt02 mimics VEGF activity, targeting endothelial cell VEGFR-1/2 receptors, inhibiting cytokine–receptor interaction pathways, and promoting angiogenesis. Meanwhile, tFNA modulates key molecules in the Hippo signaling pathway within BMSCs, driving osteogenic differentiation and matrix mineralization. Craniofacial bone defects often result from imbalanced bone microenvironment homeostasis, leading to dysregulation of the angiogenesis–osteogenesis coupled signaling network and impaired bone regeneration. In this composite DNA hydrogel system, Apt02 only mimics the biological activity of VEGF rather than native VEGF protein. Theoretically, its pro-angiogenic efficacy is inferior to that of directly loaded native VEGF, and its in vivo vascular-inducing effect remains to be further verified. Furthermore, the effectiveness of this study has not been validated in large-animal bone defect models, and there is still a practical gap toward clinical translation. Yang et al. [199] constructed a dual-nanostructured DNA dynamic hydrogel. Using an amyloid fiber–clay nanosheet hybrid network to mimic ECM topology, it achieves hierarchical regulation of biological signals. QK peptides activate the VEGF/VWF pathway to promote angiogenesis, while clay-released Si^4+^ and Mg^2+^ ions upregulate Runx2 expression via Wnt/β-catenin and BMP/Smad signaling, promoting stem cell osteogenic differentiation and reconstructing an angiogenesis–osteogenesis coupled microenvironment, enabling precise spatiotemporal control. Featuring an ECM-biomimetic structure, this hybrid DNA hydrogel acts as a physical scaffold to support adhesion, spreading and migration of BMSCs and endothelial cells, facilitating their colonization and infiltration while mimicking the physical microenvironment of native bone growth. However, limitations remain: in vivo degradation of QK peptide compromises the stability of its pro-angiogenic effect, and ion release from clay is difficult to precisely control.

Secondary osteoporosis includes bone loss caused by metabolic disorders, diabetes-induced imbalance in osteogenesis–osteoclastogenesis, and joint damage from rheumatoid arthritis leading to OA. Systemic diseases or local lesions, such as diabetes or bone trauma, often result in secondary bone defects, with repair challenged by “pathological microenvironment disorder, osteogenesis–osteoclast imbalance, and insufficient vascular regeneration.” DNA hydrogels, with their superior properties, can modulate the bone regeneration microenvironment, integrate multimodal therapeutic functions, and provide novel biomaterial-based repair strategies for refractory bone defects at the pre-clinical research stage.

OP arises from an imbalance in bone remodeling, where osteoclast-mediated bone resorption exceeds osteoblast-mediated bone formation. Traditional hydrogels mainly exert effects through drug delivery. In contrast, DNA hydrogels can achieve bidirectional regulation of both cell types via their structure or sequence, simultaneously inhibiting osteoclast activity and promoting osteoblast differentiation, while recruiting endogenous stem cells. A study [200] reported a GelMA (EFL Biotechnology, Suzhou, China) hydrogel integrated with CH6 aptamer-functionalized tetrahedral DNA nanostructures (TDNs) for mandibular bone regeneration in osteoporotic bone defects, demonstrating efficacy in complex bone lesions. In summary, osteoporotic bone regeneration imbalance originates from disrupted interactions among osteoblasts, osteoclasts, and immune cells, which weakens cellular activity and microenvironmental support. DNA hydrogels can effectively ameliorate this pathological state, promoting coordinated recovery of bone regeneration and remodeling.

Diabetes-induced impairment of bone regeneration is primarily caused by a chronic inflammatory microenvironment triggered by hyperglycemia, leading to abnormal overexpression of matrix metalloproteinase-9 (MMP-9), which activates oxidative stress cascades and disrupts bone homeostasis. This pathological process inhibits the BMP2/Runx2 osteogenic signaling pathway and VEGF-mediated angiogenesis, while simultaneously activating the NF-κB pathway driven by pro-inflammatory factors such as TNF-α and IL-6, thereby creating a vicious cycle in the bone repair microenvironment. Jing et al. [185] developed a smart responsive PEG/DNA hydrogel, using an MMP-9-specific DNA aptamer as a molecular switch, which triggers hydrogel degradation at the peak of inflammation and precisely releases stem cell apical papilla-derived exosomes (SCAP-Exo, Cyagen Biosciences, Santa Clara, CA, USA). These exosomes, enriched with miRNA-126-5p and miRNA-150-5p, respectively target the MAPK/ERK and Wnt/β-catenin signaling axes and synergistically activate the PI3K-Akt-mTOR pathway, achieving dual repair by promoting both endothelial cell angiogenesis and osteoblast differentiation. This composite hydrogel is a polymer-native DNA hybrid system. The polymer matrix improves the mechanical properties and structural stability of the hydrogel, while DNA facilitates the modular construction of MMP-9-responsive aptamer switches to realize inflammation-microenvironment-driven controlled exosome release. Their hybridization synergistically combines mechanical performance and responsive programmability, showing great potential as a prospective carrier for exosome delivery and bone repair. This approach mitigates the vascularization–mineralization coupling defects under diabetic pathological conditions, offering a promising pre-clinical therapeutic paradigm for metabolic bone defect regeneration. Existing studies have demonstrated that the development of osteoporotic bone defects is strongly correlated with the disruption of systemic hormonal homeostasis. Reduced estrogen levels upregulate RANKL expression in osteoclast precursors and downregulate OPG secretion, disrupting the coupling balance between osteoblasts and osteoclasts, which ultimately leads to markedly enhanced bone resorption relative to bone formation. Excess glucocorticoids, by contrast, suppress Wnt protein synthesis and block the BMP signaling pathway, driving mesenchymal stem cells (MSCs) to preferentially differentiate into adipocytes and impairing their osteogenic differentiation potential [201].

In future pre-clinical studies, DNA hydrogels could be combined with gene-editing technologies and rationally designed to precisely modulate cellular gene expression, further improving the efficiency of bone tissue regeneration. By performing precise imaging of the patient’s lesion site, 3D printing technology can be used to fabricate DNA hydrogel scaffolds with specific shapes, structures, and properties according to individual patient requirements, achieving precise therapy [202]. After integration with 3D-printed polycaprolactone scaffolds, the system exhibits both mechanical support and synergistic bioactivity, achieving vascular–osteogenic coupled repair. Transcriptomic sequencing has verified its molecular targeting mechanisms, providing a precise nucleic acid-based nanotherapy strategy for bone regeneration [203].

In recent years, DNA hydrogels have attracted widespread attention in the field of bone tissue engineering. To address key issues such as impaired bone regeneration and metabolic imbalance under osteoporotic bone defects and other pathological conditions, researchers have employed functionalized loading strategies to promote osteogenesis, exert anti-inflammatory effects, and enhance angiogenesis. The specific clinical application strategies, experimental models, and outcomes are summarized in Table 3.

## 5. Limitations and Future Development of DNA Hydrogels in Bone Tissue Regeneration

Currently, various DNA hydrogels have been developed, constructed through different DNA nanostructure assembly strategies or combined with novel functional materials and elements via multiple crosslinking strategies [191]. As advanced DNA assemblies, DNA hydrogels retain the material characteristics of traditional hydrogels while integrating DNA’s unique biological functions, showing broad application potential in cutting-edge fields such as biosensing, tissue engineering, disease therapy, and protein engineering [208].

Traditional hydrogels usually struggle to mimic the multi-stage regulation process of bone healing and are often unable to fully induce osteogenic activity in practical applications, significantly limiting their guidance capability in bone tissue regeneration [43]. Hybrid DNA hydrogels based on synthetic biology possess excellent features such as programmability, biocompatibility, biodegradability, and stimuli-responsiveness due to the unique structure of DNA. One study [209] proposed a novel bioconversion approach driven by acidic DNA, directly using polymer chains from biomass DNA derived from fungi, plants, animals, and other biological resources to prepare DNA hydrogels. This overcomes the traditional reliance on precise sequencing and molecular modules, broadens raw material sources, and reduces preparation costs, providing a solid theoretical basis for applying easily scalable biological resources, such as Ganoderma (lingzhi) fungi, in bone regeneration materials. This approach is expected to solve the challenge of large-scale DNA hydrogel production and lays a foundation for its industrial and clinical application. In recent years, DNA hydrogels have emerged as promising biomaterials for promoting bone regeneration, showing enormous potential in the field of bone tissue engineering. Current research increasingly focuses on biomimetic strategies, aiming to replicate the natural structure and biochemical microenvironment of bone tissue.

The clinical prospects of DNA hydrogels are broad, but many challenges remain. At present, studies on the efficacy and mechanism of DNA hydrogels for osteoporotic bone defect repair remain at the pre-clinical stage. Most experiments are performed in rodent calvarial and alveolar bone defect models, while only a few studies adopt fetal-sheep-bone models. Long-term in vivo safety and efficacy evaluations in large animals (rabbit, goat, pig) are still absent, and no clinical trial data are available. Owing to prominent inter-species differences between small animals and humans in bone metabolism cycles, bone microarchitecture and immune response, as well as osteogenic–osteoclastic balance, the pro-osteogenic, anti-inflammatory and controlled-release effects observed in animal experiments cannot be directly translated into clinical outcomes and do not equal real clinical efficacy.

Current therapies for osteoporotic bone defects mainly include bioactive bone-tissue-engineered scaffolds, degradable metallic scaffolds, and cell- and gene-based therapies. The current preparation of DNA hydrogels is costly and complex, requiring expensive equipment and specialized techniques, making large-scale production difficult [210,211]. In terms of raw materials, synthetic DNA strands or DNA origami have been conventionally adopted, while the research trend is shifting toward naturally derived genomic DNA. Such genomic DNA can be extracted from salmon sperm, chicken blood and other biological waste streams, which delivers a more sustainable and cost-effective approach [212]. However, this material carries risks of pathogen contamination and immune rejection.

From a technical perspective, the fabrication process is governed by multiple parameters including temperature, pH value and ionic strength. Minor fluctuations in these parameters exert noticeable impacts on the mechanical properties and biological activity of hydrogels. As a result, consistent product quality across batches cannot be guaranteed during large-scale manufacturing.

Furthermore, the mechanical properties of DNA hydrogels need better alignment with those of natural bone tissue to meet the requirements of repairing bone defects in different locations, especially since clinical biomaterials should mimic the repair and tensile capabilities of human tissues [213]. Currently, although strategies such as modifying DNA sequences and incorporating nanomaterials have partially improved the mechanical properties of hydrogels, it remains difficult to fully replicate the complex mechanical characteristics of natural bone tissue. Meanwhile, DNA derived from biomass sources such as Ganoderma fungi may offer advantages over synthetic DNA in terms of lower cost and wider availability. However, the purity, integrity, and sequence information of Ganoderma DNA remain uncertain, which may affect the performance and functionality of the resulting hydrogel.

In addition, the long-term interactions between DNA hydrogels and the immune system require further investigation to rule out potential immunological risks. Although DNA hydrogels demonstrate considerable potential in promoting bone regeneration and constructing bone organoids, translating them from laboratory research to clinical application still requires a lengthy process [214]. Currently, treatments for osteoporotic bone defects (anti-resorptive drugs and anabolic agents) still face limitations in efficacy and notable long-term side effects, indicating a need for optimized clinical strategies [215]. Overcoming these obstacles is crucial for advancing the practical application of DNA hydrogels in regenerative medicine, enabling them to become feasible clinical solutions for bone repair and organoid construction. Future research should focus on addressing the challenges faced by DNA hydrogels across design, application, clinical translation, and commercialization processes. Although current materials and technologies provide important support for bone regeneration, they have not yet met the performance and safety standards required for broad clinical application. To advance this field, deep integration of materials science, biology, and engineering is necessary to overcome the critical limitations of existing bone substitute materials. In the future, integrating DNA hydrogels with cutting-edge technologies such as gene editing and 3D printing will promote personalized and intelligent approaches in bone regenerative medicine, offering new avenues for complex bone defect repair and bone organoid construction. DNA hydrogels are expected to broaden their applications in bone-disease-related research and complex bone tissue engineering, providing promising candidate therapeutic strategies for pre-clinical studies of bone defects.

## 6. Conclusions

DNA hydrogels, owing to their unique programmability, biodegradability, biocompatibility, and dynamic responsiveness, may exhibit favorable pre-clinical application potential for bone regeneration and remodeling. By regulating the adhesion, proliferation, and differentiation of osteoblasts and MSCs, DNA hydrogels activate key signaling pathways, remodel the bone repair microenvironment, and achieve coordinated control of vascularization and mineralization. Compared to traditional bone repair materials, DNA hydrogels demonstrate notable advantages in drug-controlled release, mechanical adaptability, and immune modulation.

The primary causes of osteoporotic bone defects are excessive bone resorption, limited bone formation, and reduced angiogenesis. As a scaffold for bone tissue engineering, DNA hydrogels can provide a favorable microenvironment for osteoblast growth, help maintain the balance between bone resorption and formation, and show promise for ameliorating pathological conditions in experimental models of osteoporotic bone defects.

Despite continuous advances in the design strategies of DNA hydrogels, many limitations and challenges remain, such as their application in 3D printing and gene-based technologies. Future research is expected to leverage the precise regulation of functional DNA components to achieve more efficient bone vascularization and regeneration, thereby advancing pre-clinical research progress toward clinical translation in regenerative medicine.

## Figures and Tables

**Table 1:** Performance comparison of different bone repair materials in osteoporotic bone defects.

Performance Metrics	Biocompatibility	Drug Loading Capacity	Programmable Capability	Mechanical Properties	Degradation Profile	Immunogenicity Risk	References
Pure DNA hydrogel	High biocompatibility, well integrated into the biological environment	Excellent drug loading capacity; capable of carrying different types of drugs	Programmability	Mechanical tunability, mainly dependent on structure and crosslinking	Degradable by nucleases; degradation products (nucleotides) can be further utilized by the body	Can reduce immune response risk	[58,90]
Hybrid DNA hydrogel	Excellent biocompatibility	Due to polymer hydrophilic/hydrophobic compatibility; capable of loading a broader spectrum of drugs	Programmability of molecular structure allows precise control over hydrogel assembly, disassembly, and responsiveness	Good elasticity and toughness	Highly controllable degradation behavior; degradation products can be metabolized	Low immunogenicity, though specific risks should be considered	[91,92,93]
Hydroxyapatite	Ideal biocompatibility, excellent for orthopedic implant scaffolds	High loading capacity; can serve as a drug carrier or controlled-release system	No programmability	Poor mechanical properties; addition of secondary biologically inert materials can enhance mechanical performance	Slow degradation rate; risk of residuals	Unlikely to induce immune response; high safety	[94,95,96,97]
Calcium phosphate ceramics	Outstanding biosafety	Porous structure provides large surface area, ideal for sustained drug release	No programmability	Brittle with low toughness; moderate structural support	Controllable degradation rate	Extremely low immunogenicity	[98,99]
Bioactive glass	Excellent biocompatibility	Delivery of active substances via ion doping, pore loading, or surface modification	No programmability	Compressive strength close to cortical bone; brittle	Adjustable degradation rate; degradation products are bioactive	Extremely low immunogenicity	[100]
Poly(methyl methacrylate, PMMA)	Biologically inert	Drug delivery via physical mixing or surface adsorption	No programmability	Good compressive and tensile strength, but high brittleness	Non-degradable material; long-term implantation shows no significant toxicity	Low inherent immunogenicity, while degradation fragments and residuals have potential immune risks	[101]
Cobalt–chromium alloy	Good biocompatibility	Drug release via coatings and loaded carriers	Controlled coating thickness and pore distribution enable programmable drug release	Elastic modulus higher than bone; excellent fatigue resistance	Non-degradable in vivo	Metal allergy	[102,103]
Titanium alloy	Good biocompatibility	Relatively low drug loading efficiency; drug loaded in coating form	No programmability	Excellent mechanical properties; elastic modulus mismatch with native bone tends to cause stress shielding	Biologically inert; not easily degradable	Can reduce risk of postoperative infection	[104,105,106,107]
Allogeneic bone	Poor biocompatibility, prone to immune rejection	Good drug loading performance; capable of carrying antibiotics, growth factors, etc.	No programmability	Good mechanical performance	Slow degradation rate	Severe immune response and risk of disease transmission	[108,109,110,111,112]
Autologous bone	Excellent biocompatibility, gold standard for bone grafts	Good drug loading capacity; combined with drugs to promote bone formation	No programmability	Good mechanical properties	Permanently implanted; non-degradable	Associated with minor immune response; no risk of viral or bacterial transmission	[113,114,115,116]
Natural polymer hydrogel	Favorable biocompatibility and low tendency to induce immune rejection	Extremely high drug loading efficiency	No programmability	Low modulus and weak mechanical strength	Slow and sustained degradation	Potential risk of immunogenicity	[117]
Synthetic polymer hydrogels	Poorer biocompatibility relative to natural polymeric hydrogels	Drug loading capacity depends on fabrication procedures and drug–polymer interactions.	No programmability	Low stiffness, high elasticity, favorable flexibility, tunable via crosslinking density	Extremely low degradability; degradation rate decreases with increasing crosslinking density	Non-degradable or slowly degradable synthetic polymers may remain in vivo and trigger immune responses.	[118]
Composite polymer hydrogel	Good biocompatibility	Drug loading governed by composition and structural design	No programmability	Excellent fracture toughness and anti-collapse performance	Controllable degradability	Potential immunogenicity	[119]

**Table 2:** Key signaling pathways activated by DNA hydrogel.

Signaling Pathway	Activation Mode	Target Genes	Biological Effects	References
Wnt/β-catenin signaling pathway	Wnt ligand binds to Frizzled receptor and LRP5/6 co-receptor, promoting Axin and Dvl recruitment to LRP5/6, triggering Fz phosphorylation and Axin degradation; activated Dvl inhibits GSK3β, leading to β-catenin accumulation and nuclear translocation.	*c-Myc*, *cyclin D1*, *CD44*, and numerous other genes	Promotes MSC differentiation into osteoblasts, inhibits chondrocyte and adipocyte formation, regulates osteoblast and osteocyte function, and influences bone mass increase	[153]
BMP/Smad signaling pathway	BMP-2 binds to serine/threonine kinase receptors, resulting in phosphorylation of Smad1/5/8.	Runx2, alkaline phosphatase (ALP), bone sialoprotein (BSP), osteocalcin (OCN), etc.	Enhances expression of osteoblast differentiation-related genes, promoting osteogenic differentiation and mineralization	[167]
Integrin-mediated pathway	LLP2A and LXW7 on the biomaterial surface specifically bind to cell surface integrins α4β1 and αvβ3, respectively, and activate corresponding signaling pathways.	*DLX5*, *RUNX2*, *COL1A1*, *CD144*, etc.	Promotes adhesion and proliferation of relevant cells, regulating gene expression related to bone formation and vascularization	[189]
FAK/PI3K/Akt/β-Catenin signaling pathway	Hydrogel properties promote cell adhesion, activate focal adhesion kinase (FAK), which in turn activates phosphoinositide 3-kinase (PI3K), leading to phosphorylation of protein kinase B (Akt) and maintenance of β-catenin accumulation.	*Runx2*, *Osterix*, *Col1*, *Alp*, *Ocn*, and other osteogenesis-related genes	Facilitates BMSC adhesion, proliferation, and osteogenic differentiation, enhancing vascularized bone regeneration capacity	[40]
Notch signaling pathway	Notch ligands bind to their receptors, undergo translocation, and activate target genes.	*BMP2*, *LRP5*, *VEGFA*, and other related genes	Promotes osteogenic differentiation and angiogenesis	[175]

**Table 3:** Clinical application strategies of DNA hydrogels in bone regeneration.

	Loaded Drug	Animal Model	DNA Hydrogel Preparation Method	Treatment Regimen	Evaluation Time	Results	Clinical Translation	Reference
Pure DNA Hydrogel	Silver Nanoclusters (AgNCs) and Tumor Necrosis Factor-α (TNF-α) Antibody	STZ-induced diabetic C57BL/6 mice with a mandibular incisor alveolar bone defect model (2 mm diameter, 1 mm depth)	Design single-stranded DNA to anneal into Y/L monomers, and self-assemble with AgNCs and antibodies to form a hydrogel	Locally inject the hydrogel directly into the bone defect site	Assess inflammation on day 3 post-treatment and evaluate bone regeneration on day 21	Bone defect site largely healed by day 21	Combined therapy for diabetic alveolar bone regeneration, anti-infection, and inflammation control	[59]
	Apt02 Aptamer-Modified Tetrahedral Framework Nucleic Acid (Apt02-tFNA)	Sprague-Dawley (SD) rats with a calvarial bone defect model (5 mm diameter)	Prepare DNA-acrylamide polymer and form DNA hydrogel via thermal activation of crosslinkers	Implantation of DNA hydrogel composite scaffold into rat calvarial bone defect	Evaluate bone regeneration at 4 and 8 weeks post-surgery	In vivo and in vitro experiments demonstrated favorable therapeutic outcomes	Repair of critical-size bone defects caused by trauma, tumors, etc.	[60]
	Exosomes Derived from Apical Papilla Stem Cells (SCAP-Exo)	STZ-induced diabetic SD rats with an anterior mandibular alveolar bone defect model	Thiolated DNA undergoes click reaction with polyethylene glycol (PEG) to self-assemble into an MMP-9-responsive hydrogel	Locally injected into mandibular bone defect area, adaptable to irregular bone defect shapes	Micro-CT (Skyscan 1276, Bruker MicroCT, Kontich, Flanders, Belgium) and histological analysis performed at 2 and 6 weeks post-surgery	Hydrogel significantly promotes vascularized bone regeneration in diabetic rats	Smart delivery system for targeted repair of bone defects in diabetic patients	[185]
	Cytokine Interleukin-10 (IL-10)	STZ-induced diabetic mice with mandibular incisor alveolar bone defect	Specific single-stranded DNAs are annealed and self-assembled with IL-10 to form ILGel	Locally injected with PBS hydrogel, IL-10, or ILGel	Relevant assessments performed on days 0, 3, and 21 post-surgery	ILGel promotes M2 polarization and osteogenesis, accelerating alveolar bone healing in diabetic rats	Immunotherapy for diabetic alveolar bone defects and periodontitis	[204]
	Enzyme (Catalase, CAT) and Biomimetic Mineral (EACP)	SD rats with a critical-size bone defect of 5 mm diameter	GelMA and DNA are hydrogen-bonded and photo-crosslinked to construct a double-network hydrogel	DNA hydrogel promotes bone regeneration via local implantation combined with photo-crosslinking	Subcutaneous implantation for 4 weeks; bone regeneration in cranial defects evaluated at 4 and 12 weeks	Treatment significantly enhances angiogenesis, bone mineralization, and anti-inflammatory effects, achieving rapid bone integration within 4 weeks	Bone regeneration scaffold promoting organoid formation, anti-inflammatory repair, and rapid bone integration	[205]
Hybrid DNA Hydrogel	Black Phosphorus Nanosheets (BPNSs) and Nucleic Acid Aptamer Apt19S	SD rats with a calvarial bone defect model (5 mm diameter)	Air–water emulsion templating combined with DNA self-assembly and gelatin chemical crosslinking to form a double-network structure	Hydrogel directly implanted into the bone defect site, filling and in situ curing	Bone regeneration evaluated via micro-CT and histological analysis at 2, 4, and 8 weeks post-implantation	Promotes new bone formation, efficiently recruits BMSCs, and enhances bone mineral density via calcium phosphate deposition	Smart bio-scaffold for bone defect repair, targeted stem cell recruitment, and regulation of the mineralization microenvironment	[206]
	Vascular Endothelial Growth Factor (VEGF)	6-week-old female SD rats with a calvarial defect model	Mix DNA with BPNSs and VEGF and anneal at room temperature to form the hydrogel	DNA hydrogel implanted into rat cranial bone defect	Evaluated at 4 and 8 weeks	Hydrogel scaffold promotes vascularized bone regeneration with favorable in vivo and in vitro outcomes	Demonstrates potential for bone repair, though still distant from clinical translation	[187]
	VEGF-Mimicking Peptide (QK Peptide)	Female SD rats with a calvarial bone defect model	Co-assemble amyloid fibers, clay nanosheets, and DNA, with QK peptide modification, via multi-step reactions	Hydrogel implanted into rat cranial bone defect	Evaluated at 4, 8, and 12 weeks post-surgery	Hydrogel exhibited excellent performance and significantly promoted vascularized bone regeneration	Provides a novel approach for bone repair with potential for clinical bone regeneration therapy	[199]
	Dexamethasone (DEX, Sigma-Aldrich, St. Louis, MO, United States)	SD rats with calvarial bone defect (5 mm diameter)	Heat-denatured DNA followed by cooling to achieve re-crosslinking	Minimally invasive local injection into the bone defect using a medical syringe	Assessment of new bone formation at 4 and 8 weeks postoperatively	Significantly promotes new bone volume, density, and maturation	Non-load-bearing bone defect repair, drug-controlled release carrier, promoting bone regeneration and minimally invasive treatment	[145]
	VEGF and RGD Peptide	Rat calvarial defect model	Use 8-arm PEG-NB as a backbone, mix with MMP-sensitive peptides, RGD peptides, VEGF-conjugated DNA strands, actin, and photoinitiator, then UV irradiation for crosslinking	Surgical implantation of hydrogel precursors into the bone defect	Evaluation of relevant bone parameters at 2, 6, and 12 weeks postoperatively	Enhances bone morphometric parameters and angiogenesis	Bone defect-related disorders	[207]

## Data Availability

No new data were created or analyzed in this study. Data sharing is. applicable to this article.

## Abbreviations

The following abbreviations are used in this manuscript:

MSC(s)	Mesenchymal Stem Cell(s)
BMSCs	Bone Marrow Mesenchymal Stem Cells
OP	Osteoporosis
BT	Bone Tumors
ECM	Extracellular Matrix
OC	Osteoclast
OA	Osteoarthritis
ALP	Alkaline Phosphatase
BMP	Bone Morphogenetic Protein
HIV	Human Immunodeficiency Virus
VEGF	Vascular Endothelial Growth Factor
PDGF	Platelet-Derived Growth Factor
TGF-α/β	Transforming Growth Factor-α/β
OCN	Osteocalcin
tFNAs	tetrahedral Framework Nucleic Acids
pre-OB	pre-Osteoblast
GS	Glycine–Serine
MAPK	Mitogen-Activated Protein Kinase
NIC	Notch Intracellular domain
GPCRs	G-Protein-Coupled Receptors
RTKs	Receptor Tyrosine Kinases
PDK1	Phosphoinositide-Dependent Kinase 1
TDN	Tetrahedral DNA Nanostructures
M1/2	M1/2 macrophages
mTOR	Mammalian Target of Rapamycin
Runx2	Runt-related Transcription Factor 2
PI3K	Phosphoinositide 3-Kinase
Akt	Protein Kinase B
NF-κB	Nuclear Factor Kappa-B
IL-6	Interleukin-6
IL-1β	Interleukin-1β
LRP5/6	Low-density Lipoprotein Receptor-related Protein 5/6
CK1	Casein Kinase 1
GSK3	Glycogen Synthase Kinase 3
AXin	Axis Inhibition Protein
APC	Adenomatous Polyposis Coli
DVL	Dishevelled
β-catenin	Beta-catenin
PYGO	Pygopus
LGS	Legless
PKG	Protein Kinase G
TCF/LEF	T-Cell Factor/Lymphoid Enhancer Factor
cGMP	(Cyclic) Guanosine Monophosphate
PLC	Phospholipase C
IP3	Inositol 1,4,5-Trisphosphate
HSCs	Hematopoietic Stem Cells
RANKL	Receptor Activator of Nuclear Factor κB Ligand
OPG	Osteoprotegerin
SASP	Senescence-Associated Secretory Phenotype
ROS	Reactive Oxygen Species
